# Author’s response: Peanut butter confirmed as the source of a case of infant botulism in the United Kingdom, 2024

**DOI:** 10.2807/1560-7917.ES.2025.30.40.2500757

**Published:** 2025-10-09

**Authors:** Corinne FL Amar, Rosie J Crane, Shamez Ladhani, Dunstan Rajendram, Vanessa K Wong, Gauri Godbole

**Affiliations:** 1 United Kingdom Health Security Agency (UKHSA), London, United Kingdom; 2 St George's University Hospitals NHS Foundation Trust, London, United Kingdom

**Keywords:** Infant botulism, peanut, Clostridium botulinum, allergy

**To the editor: **We thank the authors of the letter for their interest in our recent report on infant botulism linked to peanut butter [1]. We welcome the opportunity to clarify several points raised and provide additional details about the exposure.

Firstly, while we agree that *Clostridium botulinum* spores are widely present in the environment [2], however, the specific circumstances of this case support our conclusion that the peanut butter was the most likely source of exposure. The implicated product was a squeeze bottle of commercially prepared peanut butter, not a jar, thus limiting opportunities for environmental contamination. The bottle was carefully stored by the family because a family member had a severe peanut allergy. Importantly, the infant’s mother dispensed the peanut butter directly from the squeeze bottle onto a spoon before feeding, minimising handling and contact with other foods or surfaces. The small outlet of the squeeze bottle further reduces the plausibility of environmental spores contaminating the bottle after purchase.

Secondly, the infant’s stool isolate and the isolate recovered from the peanut butter bottle were indistinguishable by whole genome sequencing, providing strong molecular evidence of linkage. While it is correct that testing of unopened containers from the same batch would have strengthened the investigation, the absence of such testing does not negate the significance of a matching strain recovered from both the infant and the food consumed. In this respect, our use of the term “confirmed” is consistent with the terminology applied in prior publications where food-borne or intestinal toxaemia botulism was linked to matching isolates from patients and ingested foods [3].

Thirdly, while we agree that environmental exposure is a recognised risk factor for infant botulism, the clinical and epidemiological timeline is notable: the infant received incremental doses of peanut butter for 10 days before symptom onset, consistent with the expected incubation period. No other suspect foods or environmental exposures showed similar temporal association.

We fully support the recommendation that future investigations should, wherever possible, include unopened containers from the same production batch as well as environmental sampling to exclude potential household contamination and spore count. However, given the epidemiological evidence, careful handling practices, and the genomic identity of the isolates, we consider it reasonable and proportionate to conclude that the peanut butter was the most likely source in this case.

We agree that ongoing vigilance, combined with rigorous and standardised approaches to food sampling in suspected cases such as spore counting will be essential to further our understanding of rare but important exposures leading to infant botulism.
